# A comparison of cancellous screws in a sliding compression configuration and angle-stable sliding compression implants for internal fixation of femoral neck fractures in the non-elderly predominantly below 65 years: a systematic review and meta-analysis

**DOI:** 10.2340/17453674.2025.44034

**Published:** 2025-06-16

**Authors:** Michaela M HANSEN, Mads S NIELSEN, Per H GUNDTOFT, Maiken STILLING, Ming DING, Bjarke VIBERG

**Affiliations:** 1 Department of Orthopaedic Surgery and Traumatology, Odense University Hospital; 2 Department of Clinical Medicine, University of Southern Denmark; 3 Department of Orthopaedic Surgery and Traumatology, Aarhus University Hospital; 4 Department of Clinical Medicine, Aarhus University, Denmark

## Abstract

**Background and purpose:**

Internal fixation is the preferred treatment in the non-elderly with femoral neck fractures, regardless of fracture displacement. High complication rates are reported, in particular for displaced fractures. We aimed to compare cancellous screws with angle-stable sliding compression implants for internal fixation of femoral neck fractures in the non-elderly.

**Methods:**

A systematic search was carried out in Medline, Embase, Scopus, and Cochrane. The search results were screened by 2 reviewers using Covidence and assessed for risk of bias. All comparative studies were included. The studies reported at least 1 of the following outcomes: avascular necrosis, fixation failure/cut-out, non-union, any complication, reoperation, femoral neck shortening, or Harris Hip Score (HHS). Dichotomous outcomes are reported as risk ratio (RR) and continuous outcomes as mean difference (MD). All effect measures use a random effects model.

**Results:**

The search yielded 23 studies eligible for inclusion: 4 randomized controlled trials (RCTs) and 19 retrospective cohort trials, including 1,844 fractures. Only 1 study had low risk of bias. The results demonstrated no difference in RCTs alone. Analysis of all studies showed superior outcomes in favor of angle-stable sliding compression implants for fixation failure/cut-out (RR 0.54, 95% confidence interval [CI] 0.31–0.94), any complication (RR 0.49, CI 0.28–0.87), shortening > 5 mm (RR 0.54, CI 0.37–0.80), and HHS 6–24 months (MD 3.1, CI 1.8–4.4).

**Conclusion:**

RCTs alone showed no significant differences between implant types. When including retrospective studies, angle-stable sliding compression implants demonstrated some advantages. The strength of evidence is limited by the predominance of retrospective cohort studies and high risk of bias in the included studies.

A femoral neck fracture is a detrimental health problem, affecting not only the elderly, but the young and middle-aged as well [1]. Several studies have demonstrated that non-elderly individuals with hip fractures often have impaired bone health. In a prospective study of patients aged 20–49 years, bone mass density was significantly lower compared with age-matched controls, regardless of trauma mechanism, suggesting underlying skeletal fragility [2]. Likewise, patients under 70 years of age have a high prevalence of osteoporosis, osteopenia, and significantly lower bone density [3,4].

Internal fixation is the preferred treatment for femoral neck fractures in the non-elderly due to its long-term performance and the risk of revision of hip arthroplasty [5,6]. However, a 5-year reoperation rate of 30% is reported after internal fixation [7]. When including only displaced femoral neck fractures the risk of failure can be as high as 59% [1]. Complications such as non-union, avascular necrosis, fixation failure, and femoral neck shortening are known adverse events [8-10].

There are a variety of implants for femoral neck fractures, but no clear consensus exists regarding which implant is superior in the management of femoral neck fractures in the non-elderly [11]. Several studies have evaluated implants for internal fixation of femoral neck fractures in the elderly [12]. Systematic reviews comparing cannulated cancellous screws (CCS) with sliding hip screw (SHS) or femoral neck system (FNS) show that SHS and FNS are associated with fewer postoperative complications across all age groups [13,14]. However, there are only a few studies comparing outcomes based on the type of implant used for internal fixation in the non-elderly [15].

Aiming for evidence-based clinical decision-making regarding internal fixation of femoral neck fractures in the non-elderly, typically below 65 years of age, in a systematic review and meta-analysis we compared clinical and functional outcomes of standard CCS with angle-stable sliding compression implants.

## Methods

### Design

This study was conducted as a systematic review and meta-analysis and was reported based on the Preferred Reporting Items for Systematic reviews and Meta-Analysis Protocols (PRISMA] guidelines [16].

### Eligibility criteria

The inclusion criteria were based on the population/intervention/comparator/outcome/study design (PICOS) principle [17]:

1. Population: Adults 18–69 years of age with femoral neck fracture.
2. Intervention: Patients treated with CCS in a sliding compression configuration (triangle, inverted triangle, parallel).
3. Comparator: Patients treated with any other angle-stable sliding compression implant.
4. Outcomes: Studies that had at least 1 of the following outcomes: avascular necrosis, fixation failure/cut-out, non-union, any complication, reoperation, femoral neck shortening >5 mm, failure (not specified), pain Visual Rating Scale, Oxford Hip Score, Harris Hip Score (HHS), and 1-year mortality.
5. Study design: RCTs, non-randomized controlled trials or quasi-randomized controlled trials, prospective cohort trials or retrospective comparative studies.

Exclusion criteria were:

1. Full text not available in English, Danish, Norwegian, or Swedish.
2. Less than 10 participants.
3. Less than 6 months’ follow-up.
4. Meeting abstracts, conference proceedings, biomechanical studies, animal studies, editorials, case reports/series, reviews, letters, surveys, expert opinions, non-comparative studies.
5. Patient population with ipsilateral femur fractures.
6. Studies reporting management using open reduction and/or grafting.
7. Studies reporting delayed management and/or management of complications.
8. Studies in which the relevant data could not be extracted (e.g., only P value reported or outcome not based on type of implant).
9. Pediatric (<18 years) or elderly (≥70 years) population.

These studies would be excluded if the age of the study participants was not reported or if the age was reported only as a mean and the mean age +2 standard deviations was above 70 years of age. The cut-off at 70 years was chosen as the elderly are more likely to undergo arthroplasty rather than internal fixation, while non-elderly patients (<70 years) are more likely to benefit from preserving the native hip joint through internal fixation [6,18]. Furthermore, this age span was selected to reflect most existing studies, which typically report outcomes within this range and rarely use narrower age brackets.

In studies with mixed series, including for example both femoral neck fractures and intertrochanteric fractures or both non-elderly and elderly patients, only groups that met the inclusion criteria and had data for femoral neck fractures and/or patients <70 years of age reported separately were included in the study.

### Information sources

A systematic literature search was carried out in April 2024 in Medline, Embase, Scopus, and Cochrane. The reference lists of the studies extrapolated from full text screening were manually screened for additional eligible studies.

### Search strategy

A comprehensive search string was built under guidance of a research librarian and adjusted to each specific database, to identify studies on internal fixation of femoral neck fractures. MeSH terms and keywords related to femoral neck fractures and internal fixation were used. The search string was validated by identifying 3 preselected relevant articles and confirming that they were identified using the search string [19-21]. No terms regarding age were used, and studies with a patient population ≥70 years of age were excluded manually during screening. A full description of the search strategy for each database may be found in Supplementary data.

### Selection process

Screening was conducted using Covidence systematic review software (Veritas Health Innovation, Melbourne, Australia). The Covidence software was used to automatically remove duplicates before screening. The titles and abstracts were screened independently by reviewers MH and MN after the automatic removal of duplicates. If a title clearly did not meet the eligibility criteria, the study was excluded on title alone. If the title in any way indicated eligibility, the abstract was screened. The reviewers erred on the side of inclusivity and if the abstract did not contain enough information to exclude the study, the full text was screened. Full text screening was conducted independently by the same 2 reviewers, MH and MN, and excluded studies were classified according to the reason for exclusion. In the case of discrepancy the eligibility was discussed between the 2 reviewers and, if necessary, with the other co-authors.

### Data collection process and data items

Data was collected by 2 reviewers using a prefabricated data extraction form. The following study characteristics were extracted from the eligible studies: title, authors, publication year, journal, country, study design, target population, mean age, sample size of each group, implants used for internal fixation, and follow-up duration. The clinical outcomes extracted were: avascular necrosis, fixation failure/cut-out, non-union, any complication, reoperation, femoral neck shortening >5 mm [22], failure (not specified), pain Visual Rating Scale, Oxford Hip Score, HHS, and 1-year mortality. The functional outcomes were extracted for 6–24 months and >24 months.

### Synthesis methods

RCTs are reported both separately and pooled with retrospective cohort studies. The studies were eligible for synthesis if at least 1 of the prespecified outcomes was reported. The functional outcomes had to be reported for follow-up at either 6–24 months or >24 months. Femoral neck shortening had to be defined as >5 mm. If the shortening was not defined, it would not be included in analysis.

### Study risk of bias assessment

Risk of bias assessment was performed by 2 reviewers using Cochrane’s tools for risk of bias assessment RoB2 [23] and ROBINS-I [24]. For each domain, 3 measurements were used. Any disagreement in risk of bias assessment was resolved between the 2 reviewers.

### Reporting bias assessment

Clinical trial registers and protocols were screened to assess outcome reporting bias for the included studies. Funnel plots were used to assess publication bias for outcomes with more than 10 studies.

### Statistics

Statistical analysis was performed using RevMan v8.19.0 (https://training.cochrane.org/online-learning/core-software) [25]. Dichotomous outcomes were analyzed using inverse variance with risk ratio as the effect measure. Continuous outcomes were analyzed using inverse variance with mean difference as the effect measure. The analyses use random effect models, as the data are assumed to have heterogeneity. Heterogeneity was estimated using Restricted Maximum-Likelihood [26]. All outcomes are reported with a 95% confidence interval (CI) using a Wald-type method.

### Ethics, registration, data sharing plan, funding, and disclosures

No ethical approval was needed. The protocol was registered in PROSPERO, the international prospective register of systematic reviews (CRD42024498684). The protocol has been amended to specify the intervention/comparator as CCS compared with angle-stable sliding compression implants and not “any” implant, as the search yielded several reports on non-sliding length stable implants. There were enough reports for a secondary review focusing on internal fixation with sliding compression cannulated cancellous screws compared with non-sliding implants. The strategy for data synthesis has been updated to be more specific and described in detail prior to data synthesis.

Femoral neck shortening has been added as an outcome because it is an implied postoperative complication closely related to fixation failure and functional outcomes. While it was not explicitly registered in PROSPERO, femoral neck shortening is an intermediary outcome that may contribute to fixation failure, non-union, and poor functional scores. We believe its inclusion strengthens the analysis rather than diverging from the registered outcomes. This addition does not alter the study’s primary objectives but provides further insight.

Data is available upon reasonable request through the corresponding author.

This systematic review is part of a PhD project supported by the Region of Southern Denmark’s fund to support clinical doctoral candidates. The funder has no role in the design, analysis, data interpretation, or decision to submit results.

No competing interests are reported. Complete disclosure of interest forms according to ICMJE are available on the article page, doi: 10.2340/17453674.2025.44034

## Results

### Study selection

**Figure 1:**
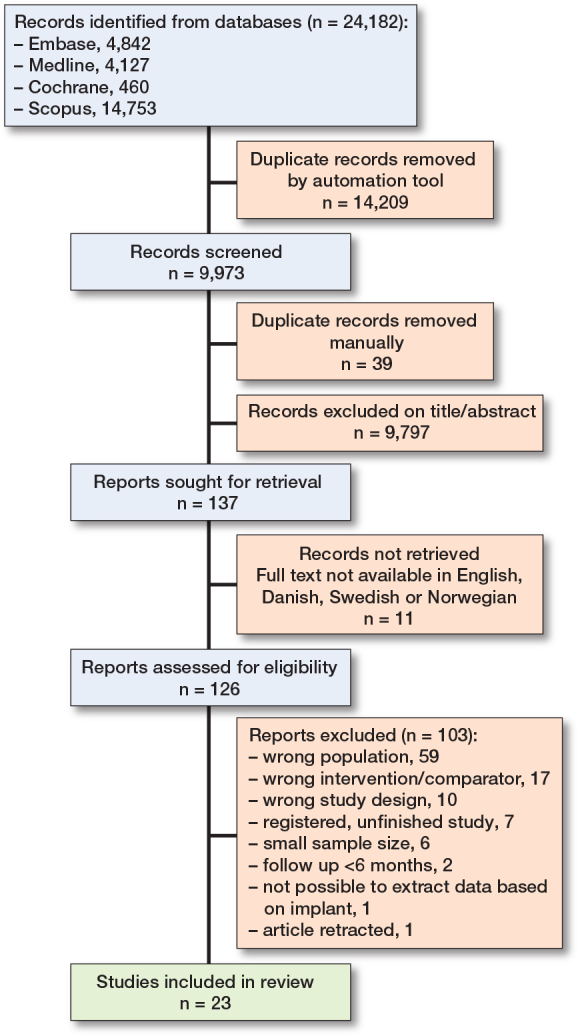
PRISMA flowchart.

9,973 reports were screened for title/abstract and 126 were full text screened. The screening yielded 23 studies [19-21,27-46] eligible for inclusion (Figure 1) [47].

Most full text screened records were excluded due to the population being ≥70 years of age or not being femoral neck fractures. Other reasons for exclusion were the study not being comparative or that the intervention or comparator did not align with the inclusion and exclusion criteria. Most studies were excluded for more than 1 reason. We identified 1 ongoing study not yet recruiting [48].

### Study characteristics

Our review includes 1,844 patients from 23 studies of which 4 are RCTs and 19 are retrospective cohort studies (Table 1). The control group consists of CCS in a sliding compression configuration. The experimental group consists of Targon Locking plate, SHS ± derotation screw, and FNS ± derotation screw.

**Table 1:** Study characteristics. In all studies patients treated with cannulated cancellous screws were control group

Reference	Design	Country	Sample size	Age	Fracture classification	Follow-up (months)	Experimental
Hegazy 2023 [32]	RCT	Egypt	50	18–60	Garden I–IV	12–36	TFN
Siavashi 2015 [39]	RCT	Iran	58	18–60	Garden III–IV	12–36	SHS + derotation screw
FAITH trial 2017 [46]	RCT	International	221	50–60	ND	24	SHS
Gupta 2016 [30]	PCS	India	85	16–60	Garden I–IV	48	SHS
He 2021 [31]	RCS	China	69	18–65	Garden I–IV	17	FNS
Hu 2021 [19]	RCS	China	44	<60	All	12	FNS
Huang 2023 [33]	RCS	China	87	<65	Garden III–IV	12	FNS
					Pauwel III		
Kenmegne 2023 [20]	RCS	China	114	18–65	All	12–36	FNS
Yan 2023 [21]	RCS	China	49	18–60	All	21–24	FNS
Zhang 2022 [44]	RCS	China	69	<65	Garden II–IV	6	FNS
Zhou 2021 [45]	RCS	China	60	<65	Pauwel III	10–22	FNS
Su 2023 [41]	RCS	China	129	18–65	All	12	FNS ± derotation screw
Cai 2024 [28]	RCS	China	120	<65	All	12	SHS and FNS
Xu 2023 [43]	RCS	China	65	18–60	All	12	SHS and FNS
Gardner 2015 [29]	RCS	USA	69	<60	Garden III–IV	11–30	SHS
Kaplan 2012 [35]	RCS	Turkey	66	18–68	Garden I–IV	7–57	SHS
Lim 2024 [36]	RCS	Singapore	57	16–64	All	12	SHS
Liporace 2008 [37]	RCS	USA	46	19–64	Pauwel III	19–36	SHS
Jiang 2021 [34]	RCS	China	139	20–60	Garden I–IV	>24	SHS + derotation screw
					Pauwel II–III		
Stockton 2019 [40]	RCS	USA	201	18–55	Garden III–IV	N/A	SHS + derotation screw
Bouaicha 2023 [27]	RCS	Tunisia	72	18–65	Garden I–IV	>24	SHS ± derotation screw
Razik 2012 [38]	RCS	UK	92	<60	Garden I–IV	24	SHS ± derotation screw
Warschawski 2021 [42]	RCS	Israel	103	<65	Garden III–IV	84	TFN

RCT: randomized controlled trial, PCS: prospective comparative study, RCS: retrospective cohort study, ND: no data, SHS: sliding hip screw, FNS: femoral neck system, TFN: Targon femoral neck locking plate

### Risk of bias in studies

The included studies are considered to have moderate to high risk of bias (Tables 2 and 3). Only 1 RCT is rated as low risk, while 2 others raise concerns regarding the randomization due to unclear methodology [30,39]. Domain 2 is inadequately reported in these 2 studies, and 1 study documents crossover without detailing deviations among participants aged 50–60 years [46]. None of the studies report missing outcome data. Bias in the measurement of outcome is considered low risk when the outcomes are clearly defined, minimizing the risk of bias.

**Table 2:**
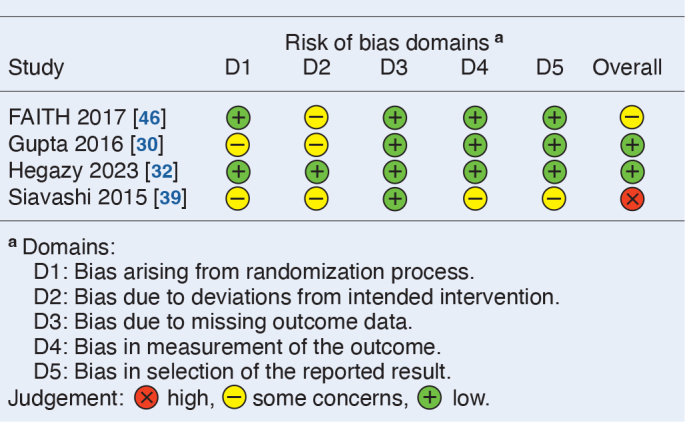
Risk of bias assessment of randomized studies, RoB 2

**Table 3:**
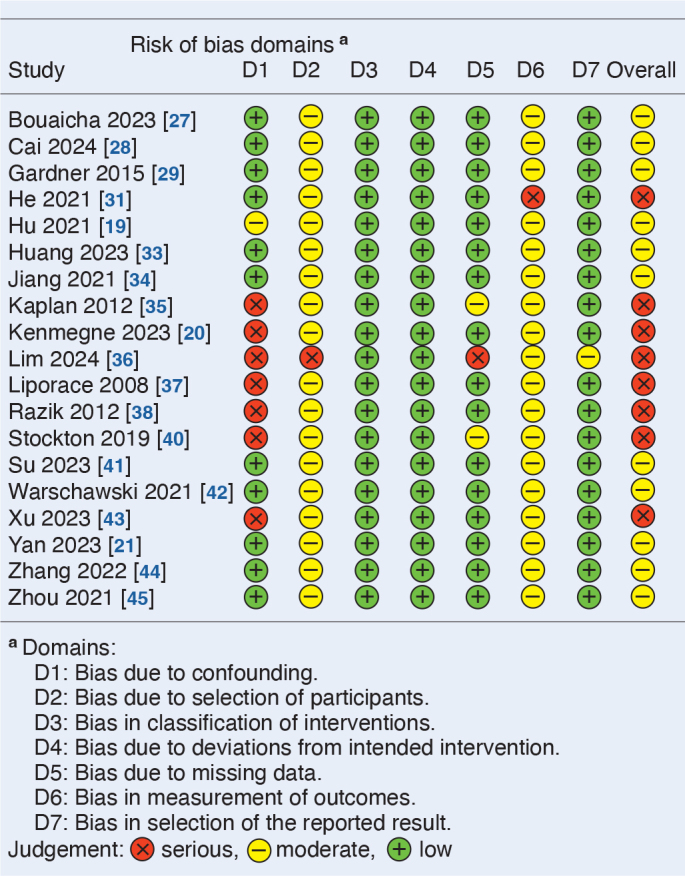
Risk of bias assessment of retrospective cohort studies, ROBINS-I

In the retrospective cohort studies, the main risks of bias are confounding, selection bias, and outcome measurement bias. Confounding arises from differences in patient characteristics such as fracture displacement, age, reduction quality, and comorbidities. Selection bias stems from non-randomized treatments, and the retrospective design inherently excluding patients with incomplete follow-up. Intervention classification is clearly defined and adherence to treatment is not considered relevant. Outcome measures are influenced by the assessor’s awareness of the intervention, resulting in moderate concerns for outcome measurement bias.

### Results of individual studies

Avascular necrosis, fixation failure, non-union, and reoperation were the only outcomes reported by RCTs. The most reported outcomes were avascular necrosis, fixation failure/cut out, and non-union, which were reported by 18, 17, and 15 studies, respectively.

None of the included studies report Oxford Hip Score, pain Visual Rating Scale, or 1-year mortality.

### Results of syntheses

**Figure 2:**
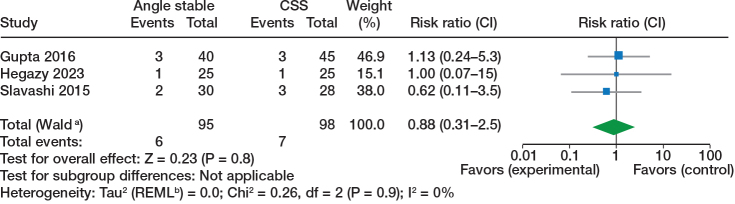
Avascular necrosis, randomized trials. Analyses used inverse variance with risk ratio as outcome with random effect models. CSS = cannulated cancellous screws, CI = 95% confidence interval. ^a^ CI calculated by Wald-type method. ^b^ Tau2 calculated by Restricted Maximum-Likelihood method.

**Figure 3:**
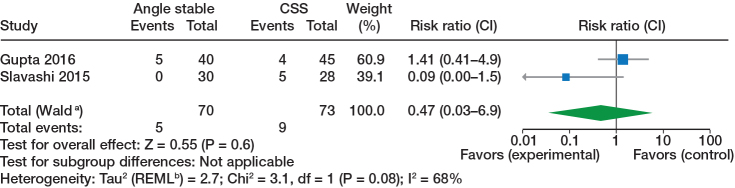
Fixation failure/cut out, randomized trials, see comments in Figure 2.

**Figure 4:**
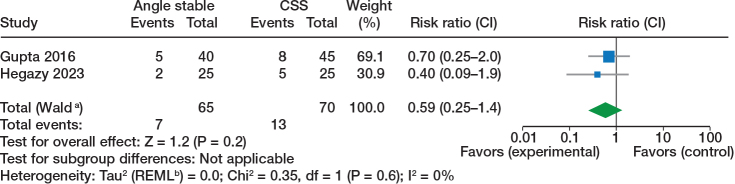
Non-union, randomized trials, see comments in Figure 2.

**Figure 5:**
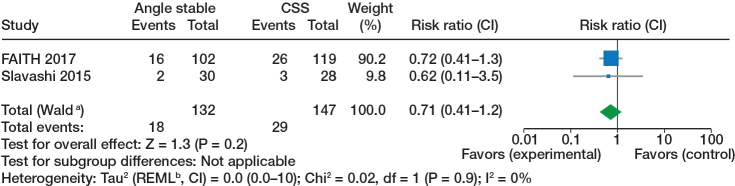
Reoperation, see comments in Figure 2.

The detailed results are shown in Table 4 and Figures 2–5. Results from combined analyses may be found in the Supplementary data. Meta-analysis on RCTs alone demonstrates no significant differences. The meta-analysis that included retrospective cohort studies shows a significant difference in risk ratio of fixation failure/cut out, any complication, and shortening >5 mm in favor of angle-stable sliding compression implants as well as a significant difference in weighted mean difference in HHS at 6–24 months in favor of angle-stable sliding compression implants. All studies reporting HHS at 6–24 months are comparisons between CCS and FNS. Cai et al. report comparisons of HHS between CCS, FNS, and SHS [28].

**Table 4:** Summary of findings

Outcomes	Sample size			Risk ratio (CI)	I^2^ %	P value
	Studies n	Angle stable	CCS			
Randomized trials						
Avascular necrosis	3	95	98	0.88 (0.31–2.5)	0	0.8
Fixation failure/cut-out	2	70	73	0.47 (0.03–6.9)	68	0.6
Non-union	2	65	70	0.59 (0.25–1.4)	0	0.2
Reoperation	2	132	147	0.71 (0.41–1.2)	0	0.2
All studies						
Avascular necrosis	18	711	752	1.0 (0.70–1.4)	12	1
Fixation failure/cut-out	17	736	794	0.54 (0.31–0.94)	33	0.03
Non-union	15	516	594	0.64 (0.39–1.1)	21	0.08
Any complication	7	233	230	0.49 (0.28–0.87)	47	0.02
Reoperation	6	336	292	0.85 (0.58–1.3)	0	0.4
Shortening > 5 mm	5	260	240	0.54 (0.37–0.80)	32	0.002
Failure, unspecified	3	156	253	0.83 (0.57–1.1)	0	0.3
Harris Hip Score ^a^						
6–24 months	8	317	310	3.1 (1.8 to 4.4) ^a^	70	<0.001
> 24 months	2	93	149	1.7 (–3.1 to 6.4) ^a^	0	0.5

a Mean difference.

### Reporting biases

Funnel plots of outcomes reported in 10 or more studies do not indicate reporting bias (see Supplementary data).

## Discussion

### General interpretation of results in context of other evidence

This systematic review and meta-analysis included 23 studies with 1,844 patients, the majority being below 65 years of age. The analysis of RCTs alone demonstrated no significant differences between CCS and angle-stable sliding compression implants for any outcome. When including retrospective cohort studies, angle-stable sliding compression implants were associated with an approximately 50% reduction in the risk of fixation failure/cut-out, overall complications, and femoral neck shortening > 5mm.

Albeit based on lower evidence, we find reduction in fixation failure/cut-out relevant as it supports the biomechanical advantage of angle-stable sliding compression implants, offering a degree of clinical confirmation for biomechanical expectations, even if causality cannot be firmly established. The increased stability and controlled dynamic compression provided by these implants likely contribute to reducing the risk of fixation failure, particularly in fractures prone to shearing forces [49,50].

Similarly, the reduction in overall complications and femoral neck shortening > 5mm aligns with existing literature, which associates shortening with inferior functional outcomes, gait abnormalities, and increased risk of osteoarthritis [10,51].

Although RCTs showed no differences in HHS, the combined analysis suggests a small improvement in HHS at 6–24 months postoperatively in favor of angle-stable sliding compression implants but not a clinically important difference [52].

It is also important to consider the heterogeneous population included in this review. While adults younger than 70 years of age may possess higher baseline physiological reserves, several studies indicate that this group does not necessarily experience better outcomes. A qualitative study highlighted persistent disability and pain years after surgery in patients under 60, in part due to rehabilitation programs geared toward older populations [53]. Furthermore, non-elderly adults who suffer hip fractures often have comorbidities and lifestyle risk factors such as smoking and alcohol use, which adversely impact both bone health and rehabilitation potential [1]. In this context, chronological age is a poor surrogate for biological age, as many patients resemble the elderly in terms of bone quality, general health, and rehabilitation potential, with only a minority being truly fit and active. Therefore, the wide age span in the included studies is less important than the underlying biological variability, which must be acknowledged to enable meaningful subgroup analyses.

### Limitations of evidence

The evidence in this review is limited by the predominance of non-randomized retrospective cohort studies, increasing risks of confounding, selection bias, and inaccurate outcome measurement [54]. Only 1 RCT is rated as low risk of bias, weakening the strength of conclusions. Although RCTs are preferred, their scarcity requires the inclusion of cohort studies for greater sample diversity and to assess complications and functional outcomes, despite inherent biases [55,56].

The use of both prospective and retrospective studies adds heterogeneity due to varying methodologies and follow-up, but this provides broader insights where RCT data ise insufficient [56].

Additionally, a key limitation is the inability to perform a subgroup analysis based on fracture displacement. While it would be ideal to separate undisplaced (Garden I–II) and displaced (Garden III–IV) fractures, the classification methods in the included studies are inconsistent. Of the 23 studies, only 5 specifically report on Garden III–IV fractures, while 14 include all fracture types. 1 study does not provide a classification, 1 includes Garden II–IV, and 2 report only on Pauwels III fractures without referencing the Garden classification. This variation in reporting prevents a reliable comparison based on displacement.

Another important limitation is the substantial biological variability within the included patient population. Although the age range (18–69 years) appears wide, the real issue lies in the heterogeneity in bone quality, comorbidities, and functional capacity across individuals, which is not adequately captured by chronological age alone. Ideally, analyses would stratify patients based on relevant biological or clinical profiles, but this was not feasible with the available studies. Therefore, the age span—and the biological variability it contains—reflects the current state of the literature rather than a methodological choice.

The methods used for measuring femoral neck shortening in the included studies lack sufficient accuracy. Incorporating radiostereometric analysis into clinical trials could allow for more reliable assessment of femoral neck shortening as well as provide high precision and detailed insights into both implant and fracture migration [57].

### Limitations of review process

Despite a systematic approach, several factors may limit comprehensiveness. Of 9,973 screened reports, only 23 meet the inclusion criteria. Manual selection by 2 reviewers, although thorough, introduce subjective judgment risks, and strict age limits (<70 years) exclude potentially relevant studies. Excluding studies with languages outside English, Danish, Norwegian, and Swedish may have introduced language bias. Funnel plot analyses are constrained to outcomes from 10 or more studies, possibly overlooking publication bias in areas with fewer studies.

### Conclusion

High-level evidence alone showed no significant differences between implant types for internal fixation of femoral neck fractures in the non-elderly. When incorporating lower quality evidence, angle-stable sliding compression implants may demonstrate some advantages in reducing fixation failure and overall complications compared with CCS. However, the strength of evidence is limited by the predominance of retrospective cohort studies, high heterogeneity of studies, and high risk of bias.

### Supplementary data

Search strings and Figures A–L are available as Supplementary data on the article homepage, doi: 10.2340/17453674.2025.44034

## Supplementary Material





## Notes

MH: conceptualization, methodology, investigation, formal analysis, and writing—original draft. MN: investigation and writing—review & editing. MD, MS, PG: methodology and writing—review & editing. BV: conceptualization, methodology, writing—review & editing, and supervision.

Handling co-editors: Cecilia Rogmark and Robin Christensen

*Acta* thanks Simo Samuli Aukusti Miettinen and Jonas Sundkvist for help with peer review of this manuscript.
