# Supplementary material for: A comparison of cancellous screws in a sliding compression configuration and angle-stable sliding compression implants for internal fixation of femoral neck fractures in the non-elderly predominantly below 65 years: a systematic review and meta-analysis
Source: Acta Orthop. 2025 Jun 16;96:443–51. doi: 10.2340/17453674.2025.44034 (PMC12168183; doi:10.2340/17453674.2025.44034)

SUPPLEMENTARY DATA

Search strategy

EMBASE
Search carried out April 18th 2024
Embase Classic+Embase <1946 to April 18, 2024>

1	"fem$ neck fracture*".mp.
2	exp femur neck fracture/ or exp femoral neck fracture/
3	1 or 2
4	exp osteosynthesis/
5	exp internal fixator/
6	exp bone screw/
7	internal fixation device*.mp.
8	device*, internal fixation.mp.
9	internal fixator*.mp.
10	osteosynthesis.mp.
11	osteosyntheses.mp.
12	screw*.mp.
13	4 or 5 or 6 or 7 or 8 or 9 or 10 or 11 or 12
14	3 and 13

Embase search: 4,842

MEDLINE
Search carried out April 18th 2024
Ovid MEDLINE(R) ALL <1946 to April 18, 2024>

1	"fem$ neck fracture*".mp.
2	exp Femoral Neck Fractures/
3	1 or 2
4	exp Fracture Fixation, Internal/
5	exp Internal Fixators/
6	exp Bone Screws/
7	internal fixation device*.mp.
8	device*, internal fixation.mp.
9	internal fixator*.mp.
10	osteosynthesis.mp.
11	osteosyntheses.mp.
12	screw*.mp.
13	4 or 5 or 6 or 7 or 8 or 9 or 10 or 11 or 12
14	3 and 13

Medline search: 4,127

COCHRANE
Search carried out April 18th 2024

ID	Search	Hits
#1	MeSH descriptor: [Femoral Neck Fractures] explode all trees	
#2	("femur neck fracture"):ti,ab,kw	
#3	("femur neck fractures"):ti,ab,kw	
#4	("femoral neck fracture"):ti,ab,kw	
#5	("femoral neck fractures"):ti,ab,kw	
#6	"femoral neck fracture"	
#7	"femoral neck fractures"	
#8	"femur neck fracture"	
#9	"femur neck fractures"	
#10	#1 OR #2 OR #3 OR #4 OR #5 OR #6 OR #7 OR #8 OR #9	

#11 MeSH descriptor: [Internal Fixators] explode all trees
#12 MeSH descriptor: [Fracture Fixation, Internal] explode all trees
#13 ("internal fixation"):ti,ab,kw
#14 ("Internal fixator"):ti,ab,kw
#15 ("internal fixators"):ti,ab,kw
#16 ("osteosynthesis"):ti,ab,kw
#17 ("osteosyntheses"):ti,ab,kw
#18 (screw):ti,ab,kw
#19 (screws):ti,ab,kw
#20 (implant):ti,ab,kw
#21 (implants):ti,ab,kw
#22 "internal fixation"
#23 "internal fixator"
#24 "internal fixators"
#25 osteosynthesis
#26 osteosyntheses
#27 screw*
#28 implant*
#29 #11 OR #12 OR #13 OR #14 OR #15 OR #16 OR #17 OR #18 OR #19 OR #20 OR #21 OR #22 OR #23 OR #24 OR #25 OR #26 OR #27 OR #28
#30 #10 AND #29
Cochrane search: 460

SCOPUS
Search carried out April 18th 2024
((TITLE-ABS-KEY ("femur neck fracture*" OR "femoral neck fracture*" OR "collum femoris fracture*")) OR "femur neck fracture*" OR "femoral neck fracture*" OR "collum femoris fracture*") AND ((TITLE-ABS-KEY ("internal fixation" OR "internal fixator*" OR osteosynthesis OR osteosyntheses OR screw* OR implant*)) OR "internal fixation" OR "internal fixator*" OR osteosynthesis OR osteosyntheses OR screw* OR implant*)
Scopus search: 14,753

Total reports: 24,182

Analysis of combined studies and funnel plots

Figure A. Avascular necrosis, combined studies

Study or Subgroup	Angle stable sliding compression Events	Angle stable sliding compression Total	CCS Events	CCS Total	Weight	Risk ratio IV, Random, 95% CI
Bouaicha 2023	7	39	3	33	6.7%	1.97 [0.55 , 7.03]
Cai 2024	9	80	4	40	8.4%	1.13 [0.37 , 3.43]
Gardner 2015	5	40	2	29	4.6%	1.81 [0.38 , 8.70]
Gupta 2016	3	40	3	45	4.8%	1.13 [0.24 , 5.26]
Hegazy 2023	1	25	1	25	1.6%	1.00 [0.07 , 15.12]
Hu 2021	1	20	3	24	2.5%	0.40 [0.05 , 3.55]
Huang 2023	1	42	2	45	2.1%	0.54 [0.05 , 5.69]
Jiang 2021	10	32	30	107	20.8%	1.11 [0.61 , 2.02]
Kaplan 2012	10	33	6	33	12.1%	1.67 [0.68 , 4.06]
Kenmegne 2023	1	56	4	58	2.5%	0.26 [0.03 , 2.25]
Lim 2024	4	29	1	28	2.6%	3.86 [0.46 , 32.46]
Razik 2012	1	42	12	50	3.0%	0.10 [0.01 , 0.73]
Siavashi 2015	2	30	3	28	3.9%	0.62 [0.11 , 3.45]
Su 2023	4	57	14	72	9.2%	0.36 [0.13 , 1.04]
Warschawski 2021	15	61	6	42	12.7%	1.72 [0.73 , 4.07]
Yan 2023	0	22	1	27	1.2%	0.41 [0.02 , 9.50]
Zhang 2022	0	33	0	36		Not estimable
Zhou 2021	0	30	1	30	1.2%	0.33 [0.01 , 7.87]
**Total (Wald[a])**		**711**		**752**	**100.0%**	**1.00 [0.70 , 1.43]**
Total events:	74		96			

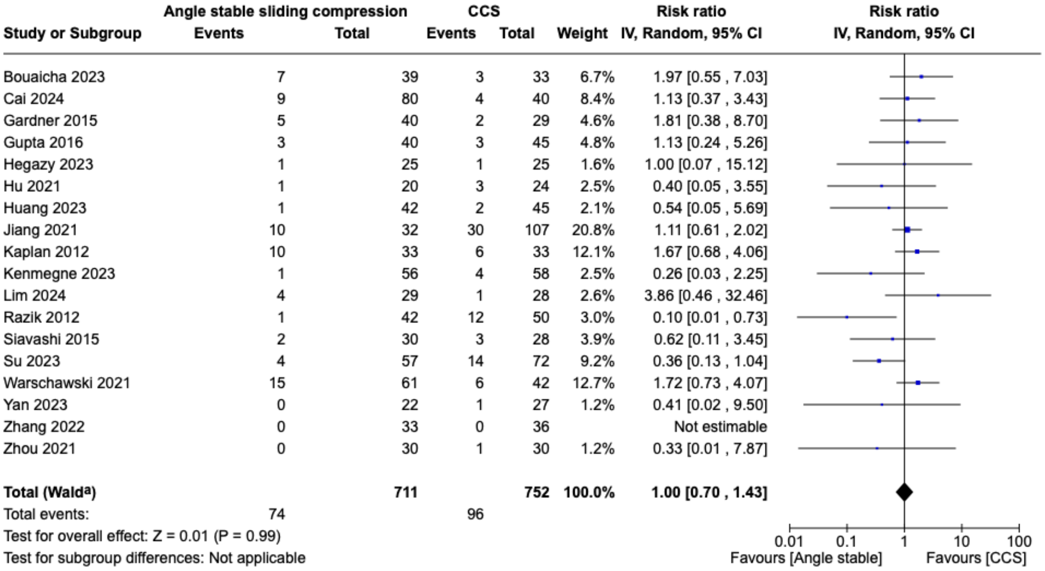

Test for overall effect: Z = 0.01 (P = 0.99)
Test for subgroup differences: Not applicable
Heterogeneity: Tau² (REML[b]) = 0.06; Chi² = 18.38, df = 16 (P = 0.30); I² = 12%

Footnotes
[a]CI calculated by Wald-type method.
[b]Tau² calculated by Restricted Maximum-Likelihood method.

Figure B. Fixation failure/cut out, combined studies

Study or Subgroup	Angle stable sliding compression Events	Angle stable sliding compression Total	CCS Events	CCS Total	Weight	Risk ratio IV, Random, 95% CI
Bouaicha 2023	2	39	2	33	5.8%	0.85 [0.13 , 5.68]
Cai 2024	5	80	2	40	7.4%	1.25 [0.25 , 6.16]
Gupta 2016	5	40	4	45	9.7%	1.41 [0.41 , 4.88]
He 2021	1	33	2	36	4.3%	0.55 [0.05 , 5.74]
Hu 2021	0	20	6	24	3.2%	0.09 [0.01 , 1.53]
Huang 2023	0	42	8	45	3.2%	0.06 [0.00 , 1.06]
Jiang 2021	3	32	10	107	9.8%	1.00 [0.29 , 3.43]
Kaplan 2012	2	33	2	33	5.9%	1.00 [0.15 , 6.68]
Kenmegne 2023	2	56	5	58	7.4%	0.41 [0.08 , 2.05]
Siavashi 2015	0	30	5	28	3.1%	0.09 [0.00 , 1.47]
Stockton 2019	6	84	2	117	7.5%	4.18 [0.86 , 20.20]
Su 2023	1	57	18	72	5.5%	0.07 [0.01 , 0.51]
Warschawski 2021	4	61	6	42	10.0%	0.46 [0.14 , 1.53]
Xu 2023	2	44	1	21	4.3%	0.95 [0.09 , 9.94]
Yan 2023	1	22	8	27	5.5%	0.15 [0.02 , 1.13]
Zhang 2022	0	33	5	36	3.1%	0.10 [0.01 , 1.72]
Zhou 2021	1	30	2	30	4.3%	0.50 [0.05 , 5.22]
**Total (Wald[a])**		**736**		**794**	**100.0%**	**0.54 [0.31 , 0.94]**
Total events:	35		88			

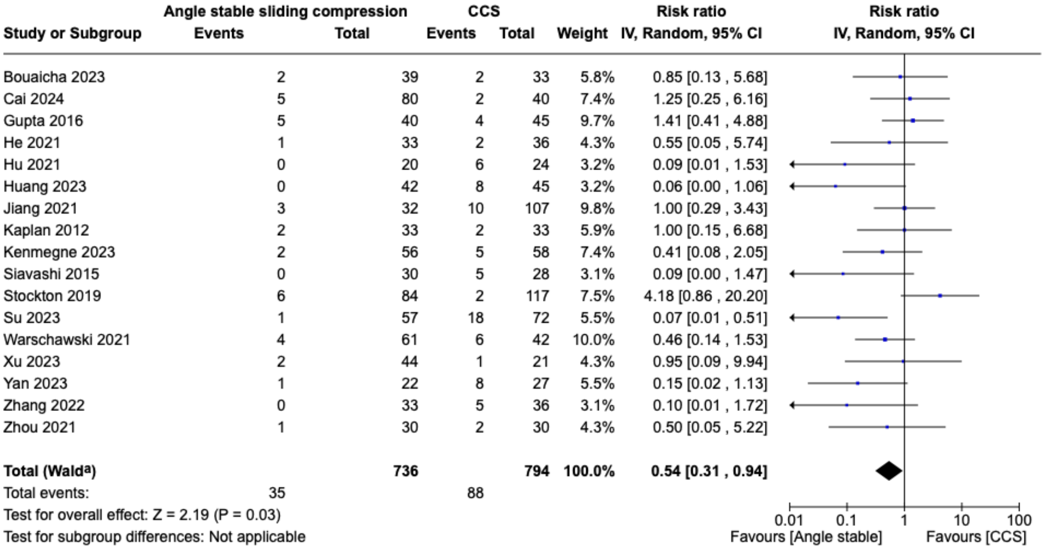

Test for overall effect: Z = 2.19 (P = 0.03)
Test for subgroup differences: Not applicable
Heterogeneity: Tau² (REML[b]) = 0.42; Chi² = 23.87, df = 16 (P = 0.09); I² = 33%

Footnotes
[a]CI calculated by Wald-type method.
[b]Tau² calculated by Restricted Maximum-Likelihood method.

Figure C. Non-union, combined studies

Study or Subgroup	Angle stable sliding compression Events	Angle stable sliding compression Total	CCS Events	CCS Total	Weight	Risk ratio IV, Random, 95% CI
Bouaicha 2023	5	39	2	33	7.6%	2.12 [0.44 , 10.20]
Gardner 2015	4	40	1	29	4.6%	2.90 [0.34 , 24.61]
Gupta 2016	5	40	8	45	13.6%	0.70 [0.25 , 1.98]
He 2021	0	33	2	36	2.5%	0.22 [0.01 , 4.37]
Hegazy 2023	2	25	5	25	7.8%	0.40 [0.09 , 1.87]
Hu 2021	2	20	3	24	6.8%	0.80 [0.15 , 4.33]
Huang 2023	1	42	4	45	4.6%	0.27 [0.03 , 2.30]
Jiang 2021	4	32	13	107	13.3%	1.03 [0.36 , 2.94]
Kaplan 2012	3	33	1	33	4.3%	3.00 [0.33 , 27.38]
Kenmegne 2023	2	56	5	58	7.4%	0.41 [0.08 , 2.05]
Lim 2024	1	29	0	28	2.3%	2.90 [0.12 , 68.33]
Liporace 2008	1	14	4	32	4.8%	0.57 [0.07 , 4.66]
Warschawski 2021	4	61	15	42	13.6%	0.18 [0.07 , 0.51]
Yan 2023	0	22	1	27	2.3%	0.41 [0.02 , 9.50]
Zhou 2021	1	30	3	30	4.4%	0.33 [0.04 , 3.03]
**Total (Wald[a])**		**516**		**594**	**100.0%**	**0.64 [0.39 , 1.05]**
Total events:	35		67			

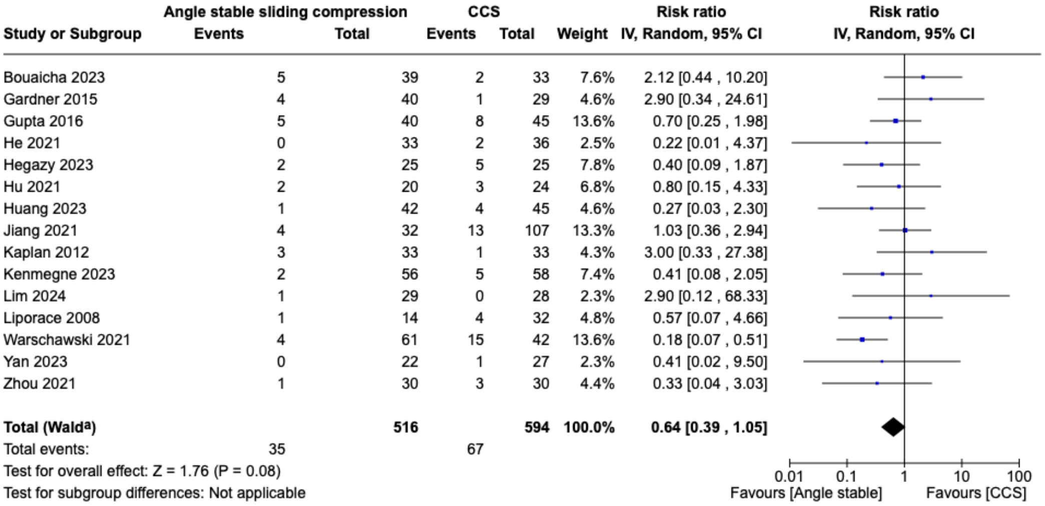

Test for overall effect: Z = 1.76 (P = 0.08)
Test for subgroup differences: Not applicable
Heterogeneity: Tau² (REML[b]) = 0.19; Chi² = 15.59, df = 14 (P = 0.34); I² = 21%

Footnotes
[a]CI calculated by Wald-type method.
[b]Tau² calculated by Restricted Maximum-Likelihood method.

Figure D. Any complication, combined studies

Study or Subgroup	Angle stable sliding compression Events	Angle stable sliding compression Total	CCS Events	CCS Total	Weight	Risk ratio IV, Random, 95% CI
Gardner 2015	10	40	9	29	20.9%	0.81 [0.38 , 1.73]
He 2021	2	33	9	36	10.6%	0.24 [0.06 , 1.04]
Hegazy 2023	4	25	6	25	14.4%	0.67 [0.21 , 2.08]
Hu 2021	3	20	12	24	14.7%	0.30 [0.10 , 0.92]
Kenmegne 2023	8	56	26	58	22.1%	0.32 [0.16 , 0.64]
Lim 2024	8	29	1	28	6.6%	7.72 [1.03 , 57.82]
Zhou 2021	2	30	9	30	10.7%	0.22 [0.05 , 0.94]
**Total (Wald[a])**		**233**		**230**	**100.0%**	**0.49 [0.28 , 0.87]**
Total events:	37		72			

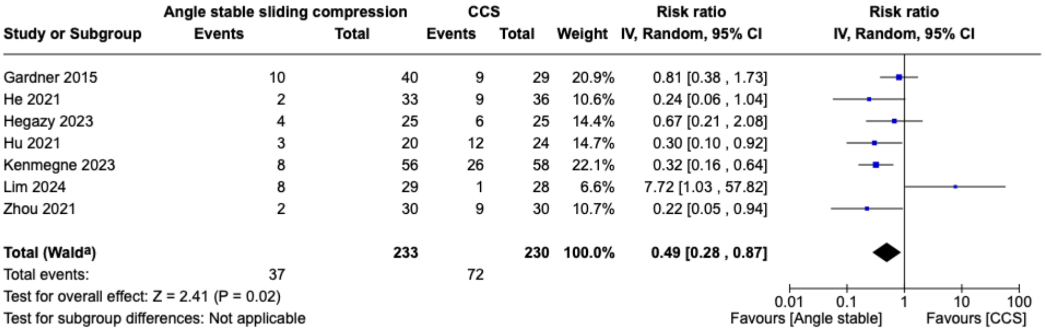

Test for overall effect: Z = 2.41 (P = 0.02)
Test for subgroup differences: Not applicable
Heterogeneity: Tau² (REML[b]) = 0.26; Chi² = 13.32, df = 6 (P = 0.04); I² = 47%

Footnotes
[a]CI calculated by Wald-type method.
[b]Tau² calculated by Restricted Maximum-Likelihood method.

Figure E. Reoperation, combined studies

Study or Subgroup	Angle stable sliding compression Events	Total	CCS Events	Total	Weight	Risk ratio IV, Random, 95% CI
Cai 2024	14	80	6	40	19.8%	1.17 [0.48 , 2.81]
FAITH 2017	16	102	26	119	48.1%	0.72 [0.41 , 1.26]
Kaplan 2012	5	33	3	33	8.4%	1.67 [0.43 , 6.41]
Siavashi 2015	2	30	3	28	5.2%	0.62 [0.11 , 3.45]
Warschawski 2021	9	61	5	42	14.7%	1.24 [0.45 , 3.44]
Zhou 2021	1	30	8	30	3.8%	0.13 [0.02 , 0.94]
**Total (Wald[a])**		**336**		**292**	**100.0%**	**0.85 [0.58 , 1.26]**
Total events:	47		51			

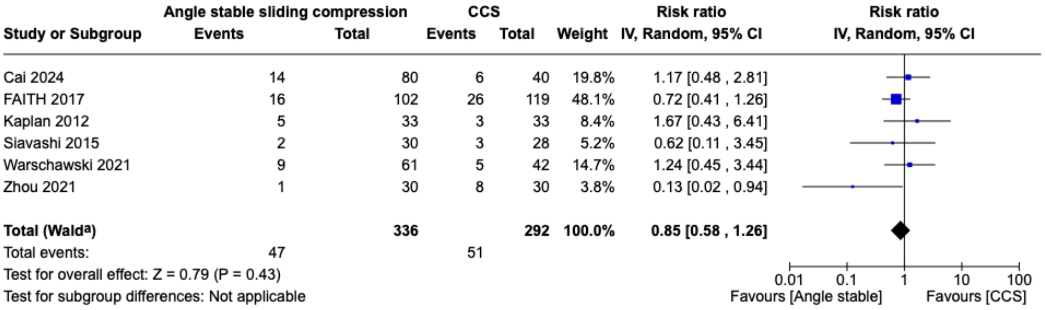

Test for overall effect: Z = 0.79 (P = 0.43)
Test for subgroup differences: Not applicable
Heterogeneity: Tau² (REML[b], 95% CI) = 0.00 [0.00 , 4.35]; Chi² = 5.93, df = 5 (P = 0.31); I² = 0%

Footnotes
[a]CI calculated by Wald-type method.
[b]Tau² calculated by Restricted Maximum-Likelihood method.

Figure F. Shortening >5mm, combined studies

Study or Subgroup	Angle stable sliding compression Events	Total	CCS Events	Total	Weight	Risk ratio IV, Random, 95% CI
Cai 2024	17	80	9	40	20.0%	0.94 [0.46 , 1.93]
Hegazy 2023	9	25	13	25	22.9%	0.69 [0.36 , 1.32]
Huang 2023	7	42	14	45	16.9%	0.54 [0.24 , 1.20]
Kenmegne 2023[a]	3	56	12	58	8.7%	0.26 [0.08 , 0.87]
Su 2023	14	57	45	72	31.4%	0.39 [0.24 , 0.64]
**Total (Wald[b])**		**260**		**240**	**100.0%**	**0.54 [0.37 , 0.80]**
Total events:	50		93			

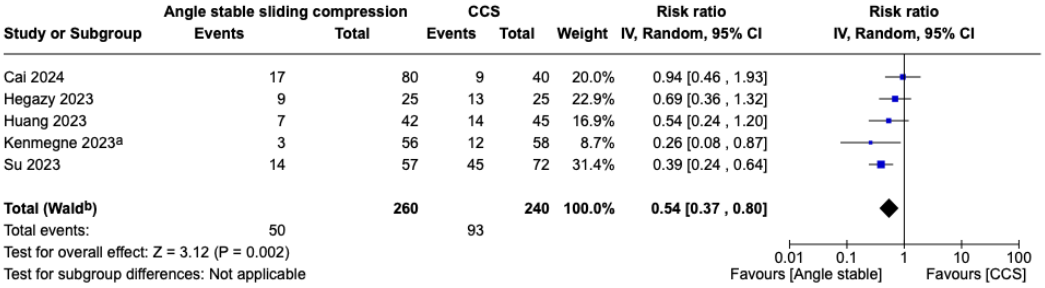

Test for overall effect: Z = 3.12 (P = 0.002)
Test for subgroup differences: Not applicable
Heterogeneity: Tau² (REML[c]) = 0.06; Chi² = 5.96, df = 4 (P = 0.20); I² = 32%

Footnotes
[a]>10mm
[b]CI calculated by Wald-type method.
[c]Tau² calculated by Restricted Maximum-Likelihood method.

Figure G. Failure, not specified, combined studies

Study or Subgroup	Angle stable sliding compression Events	Total	CCS Events	Total	Weight	Risk ratio IV, Random, 95% CI
Gardner 2015	1	40	6	29	3.1%	0.12 [0.02 , 0.95]
Jiang 2021	12	32	42	107	52.2%	0.96 [0.58 , 1.58]
Stockton 2019	16	84	28	117	44.7%	0.80 [0.46 , 1.37]
**Total (Wald[a])**		**156**		**253**	**100.0%**	**0.83 [0.57 , 1.19]**
Total events:	29		76			

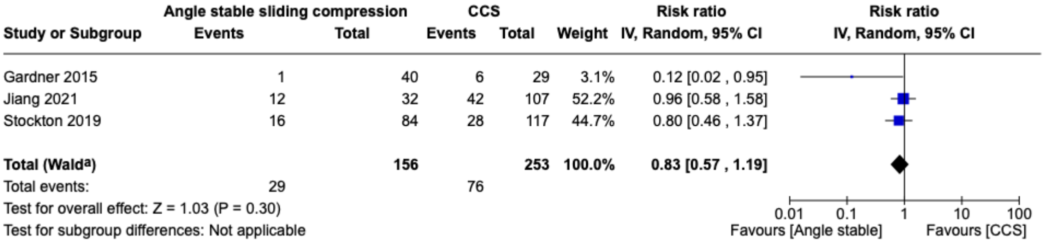

Test for overall effect: Z = 1.03 (P = 0.30)
Test for subgroup differences: Not applicable
Heterogeneity: Tau² (REML[b]) = 0.00; Chi² = 3.67, df = 2 (P = 0.16); I² = 0%

Footnotes
[a]CI calculated by Wald-type method.
[b]Tau² calculated by Restricted Maximum-Likelihood method.

Figure H. Harris Hip Score 6–24 months, combined studies

Study or Subgroup	Angle stable sliding compression Mean	SD	Total	CCS Mean	SD	Total	Weight	Mean difference IV, Random, 95% CI
Cai 2024	91.57	3.752493	80	91.03	4.2	40	16.0%	0.54 [-1.00 , 2.08]
He 2021	90.42	4.82	33	88.44	5.91	36	11.8%	1.98 [-0.56 , 4.52]
Hu 2021	85.9	5.98	20	81.92	8.34	24	6.7%	3.98 [-0.26 , 8.22]
Huang 2023	81.1	4.6	42	77.4	3.6	45	15.1%	3.70 [1.96 , 5.44]
Su 2023	87.19	2.83	57	84.03	3.41	72	18.0%	3.16 [2.08 , 4.24]
Yan 2023	90.07	5.54	22	88.18	6.19	27	9.1%	1.89 [-1.40 , 5.18]
Zhang 2022	84.61	3.42	33	78.67	3.72	36	15.4%	5.94 [4.26 , 7.62]
Zhou 2021	86.16	7.26	30	82.37	7.52	30	7.9%	3.79 [0.05 , 7.53]
**Total (Wald[a])**			**317**			**310**	**100.0%**	**3.10 [1.76 , 4.44]**

Mean difference IV, Random, 95% CI

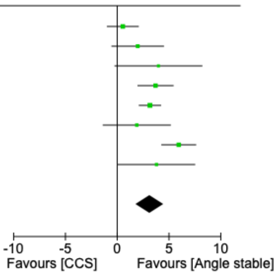

Test for overall effect: Z = 4.53 (P < 0.00001)
Test for subgroup differences: Not applicable
Heterogeneity: Tau² (REML[b]) = 2.30; Chi² = 23.57, df = 7 (P = 0.001); I² = 70%

Footnotes
[a]CI calculated by Wald-type method.
[b]Tau² calculated by Restricted Maximum-Likelihood method.

Figure I. Harris Hip Score >24 months, combined studies

Study or Subgroup	Angle stable sliding compression Mean	SD	Total	CCS Mean	SD	Total	Weight	Mean difference IV, Random, 95% CI
Jiang 2021	82.2	14.3	32	80.2	14.7	107	70.7%	2.00 [-3.68 , 7.68]
Warschawski 2021	81	23	61	80.2	22.1	42	29.3%	0.80 [-8.03 , 9.63]
**Total (Wald[a])**			**93**			**149**	**100.0%**	**1.65 [-3.13 , 6.43]**

Mean difference IV, Random, 95% CI

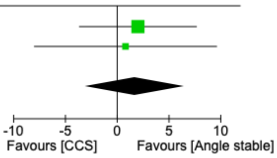

Test for overall effect: Z = 0.68 (P = 0.50)
Test for subgroup differences: Not applicable
Heterogeneity: Tau² (REML[b]) = 0.00; Chi² = 0.05, df = 1 (P = 0.82); I² = 0%

Footnotes
[a]CI calculated by Wald-type method.
[b]Tau² calculated by Restricted Maximum-Likelihood method.

Figure J. Funnel plot avascular necrosis, combined studies

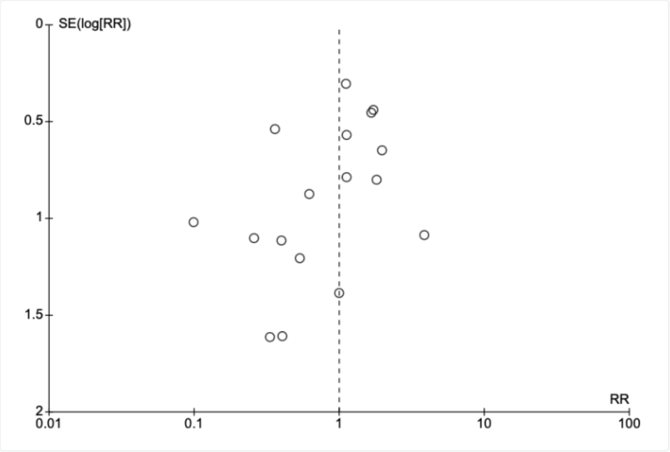

Figure K. Funnel plot fixation failure/cut out, combined studies

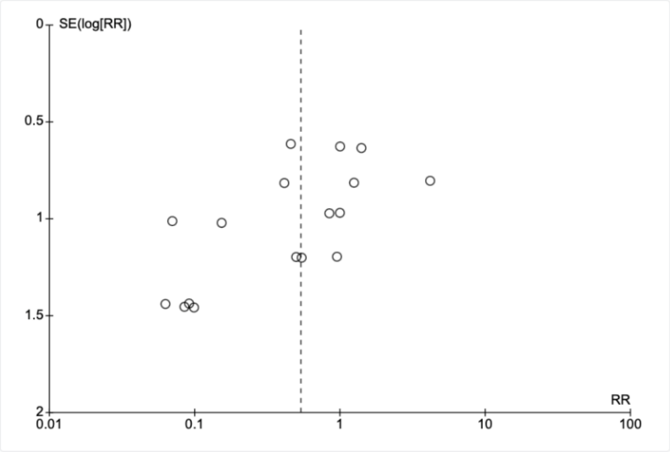

Figure L. Funnel plot non-union, combined studies

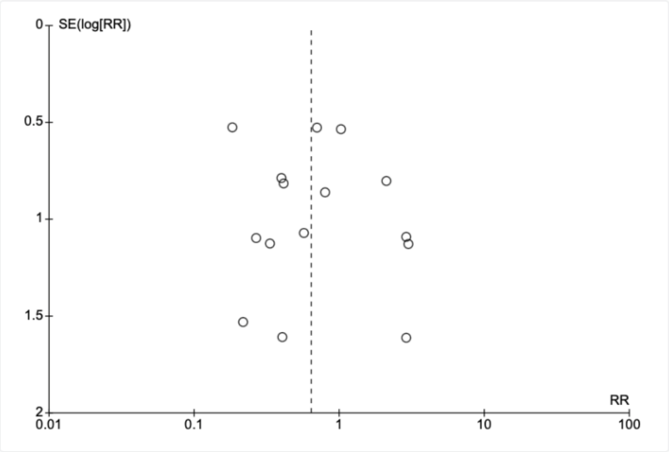

Supplement: Supplementary file 2 [file ActaO-96-44034-s2.pdf]
